# Another level of complex-ity: The role of metabolic channeling and metabolons in plant terpenoid metabolism

**DOI:** 10.3389/fpls.2022.954083

**Published:** 2022-08-10

**Authors:** Michael Gutensohn, Erin Hartzell, Natalia Dudareva

**Affiliations:** ^1^Division of Plant and Soil Sciences, West Virginia University, Morgantown, WV, United States; ^2^Department of Biochemistry, Purdue University, West Lafayette, IN, United States; ^3^Department of Horticulture and Landscape Architecture, Purdue University, West Lafayette, IN, United States; ^4^Purdue Center for Plant Biology, Purdue University, West Lafayette, IN, United States

**Keywords:** terpenoids, metabolic channeling, metabolons, prenyltransferases, membrane complexes, chlorophyll, sterols, metabolic engineering

## Abstract

Terpenoids constitute one of the largest and most diverse classes of plant metabolites. While some terpenoids are involved in essential plant processes such as photosynthesis, respiration, growth, and development, others are specialized metabolites playing roles in the interaction of plants with their biotic and abiotic environment. Due to the distinct functions and properties of specific terpenoid compounds, there is a growing interest to introduce or modify their production in plants by metabolic engineering for agricultural, pharmaceutical, or industrial applications. The MVA and MEP pathways and the prenyltransferases providing the general precursors for terpenoid formation, as well as the enzymes of the various downstream metabolic pathways leading to the formation of different groups of terpenoid compounds have been characterized in detail in plants. In contrast, the molecular mechanisms directing the metabolic flux of precursors specifically toward one of several potentially competing terpenoid biosynthetic pathways are still not well understood. The formation of metabolons, multi-protein complexes composed of enzymes catalyzing sequential reactions of a metabolic pathway, provides a promising concept to explain the metabolic channeling that appears to occur in the complex terpenoid biosynthetic network of plants. Here we provide an overview about examples of potential metabolons involved in plant terpenoid metabolism that have been recently characterized and the first attempts to utilize metabolic channeling in terpenoid metabolic engineering. In addition, we discuss the gaps in our current knowledge and in consequence the need for future basic and applied research.

## Introduction

**FIGURE 1:**
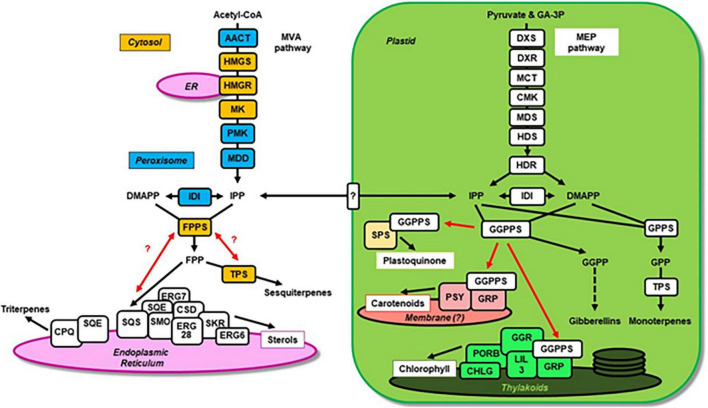
The plant terpenoid metabolic network and potential metabolons involved in the biosynthesis of specific compounds. The plastid and endoplasmic reticulum are highlighted in green and purple, respectively. The MVA pathway enzymes localized in peroxisomes and the cytosol are labeled in blue and orange, respectively. Individual enzymes are depicted as boxes and black arrows indicate metabolic fluxes. Confirmed and putative (with question mark) interactions of prenyltransferases with downstream enzymes forming metabolons are indicated by red arrows. The unknown transporter involved in IPP and DMAPP exchange between cytosol and plastid is shown in the plastid envelope membrane. Abbreviations: AACT, aceto-acetyl-CoA thiolase; CHLG, chlorophyll synthase; CMK, 4-(cytidine 5-diphospho)-2-C-methyl-D-erythritol kinase; CPQ, cucurbitadienol synthase; CSD, sterol C-3 dehydrogenase/C-4 decarboxylase; DMAPP, dimethylallyl diphosphate; DXR, 1-deoxy-D-xylulose 5-phosphate reductoisomerase; DXS, 1-deoxy-D-xylulose 5-phosphate synthase; FPPS, farnesyl diphosphate synthase; GA-3P, D-glyceraldehyde 3-phosphate; GGPPS, geranylgeranyl diphosphate synthase; GGR, geranylgeranyl reductase; GPPS, geranyl diphosphate synthase; GRP, GGPPS recruiting protein; HDR, (*E*)-4-hydroxy-3-methylbut-2-enyl diphosphate reductase; HDS, (*E*)-4-hydroxy-3-methylbut-2-enyl diphosphate synthase; HMGR, 3-hydroxy-3-methylglutaryl-CoA reductase; HMGS, 3-hydroxy-3-methylglutaryl-CoA synthase; IDI, isopentenyl diphosphate isomerase; IPP, isopentenyl diphosphate; LIL3; light-harvesting-like protein 3; MCT, 2-C-methyl-D-erythritol 4-phosphate cytidylyltransferase; MDD, mevalonate diphosphate decarboxylase; MDS, 2-C-methyl-D-erythritol 2,4-cyclodiphosphate synthase; MK, mevalonate kinase; PMK, phosphomevalonate kinase; PORB, protochlorophyllide oxidoreductase; PSY, phytoene synthase; SKR, sterol C-3 keto-reductase; SMO, sterol C-4 methyl oxidase; SPS, solanesyl diphosphate synthase; SQE, squalene epoxidase; SQS, squalene synthase; TPS, terpene synthases (including mono- and sesquiterpene synthases).

Terpenoids represent a prominent class of metabolites present in all living organisms. An exceptionally large and structurally diverse number of terpenoids is produced by plants, which employ multiple biosynthetic pathways frequently acting in parallel to create a plethora of these metabolites (Chen et al., 2011; Pichersky and Raguso, 2018). Some terpenoids found in all plants serve essential roles and include pigments (chlorophylls, carotenoids), electron carriers (plastoquinone, ubiquinone), membrane components (sterols), and hormones (gibberellins, abscisic acid, steroids, strigolactones). In contrast, the majority of plant terpenoids, in particular mono-, sesqui- and di-terpenes, are species-specific specialized metabolites that are involved in antagonistic and beneficial interactions with the environment. Due to their roles in defense against pests and pathogens, attraction of beneficial organisms, and contribution to the aromas of fruits and other edible parts of plants, these terpenoids are valuable agronomic traits. In addition, terpenoids are widely used by humans as flavors, fragrances, preservatives, pharmaceuticals and biofuels (Ajikumar et al., 2008; Immethun et al., 2013; Tippmann et al., 2013). Despite the tremendous diversity of terpenoids, they are all derived from the same precursors, isopentenyl diphosphate (IPP) and dimethylallyl diphosphate (DMAPP). In plants these isomers are synthesized by two alternative pathways (Figure 1), the mevalonic acid (MVA) pathway operating in the cytosol and peroxisomes, and the methylerythritol phosphate (MEP) pathway in plastids (Ashour et al., 2010; Hemmerlin et al., 2012). Although these pathways act independently, there is an IPP and DMAPP exchange between them *via* yet unidentified transporters in the plastid envelope membranes (Soler et al., 1993; Bick and Lange, 2003; Flügge and Gao, 2005). IPP and DMAPP are subsequently utilized by short-chain prenyltransferases that join the isoprene units in the *trans* configuration to form larger prenyl diphosphate intermediates, which ultimately serve as precursors for the downstream terpenoid biosynthesis (Figure 1). Farnesyl diphosphate synthase (FPPS) forms *trans*-farnesyl diphosphate (*E*,*E*-FPP) in the cytosol, while geranyl diphosphate synthase (GPPS) and geranylgeranyl diphosphate synthase (GGPPS) are responsible for respective geranyl diphosphate (GPP) and geranylgeranyl diphosphate (GGPP) formation primarily in plastids (Gutensohn et al., 2013). These prenyl diphosphates are then utilized by a large family of terpene synthases (TPSs) to produce the variety of mono-, sesqui- and di-terpenes in plants (Chen et al., 2011; Karunanithi and Zerbe, 2019). In parallel *E*,*E*-FPP and GGPP are used for head-to-head condensations by squalene synthase (SQS) and phytoene synthase (PSY) to form the backbones of tri- and tetra-terpenes such as sterols and carotenoids, respectively. In plastids GGPP also serves as precursor for the formation of gibberellins and the side chains of chlorophylls, tocopherols and plastoquinones (Ruiz-Sola et al., 2016). An additional class of species-specific *cis*-prenyltransferases has been identified (Oh et al., 2000; Asawatreratanakul et al., 2003; Akhtar et al., 2013), which link isoprene units in the *cis* configuration to synthesize neryl diphosphate (NPP) and *cis*-farnesyl diphosphate (*Z*,*Z*-FPP), the precursors for mono- and sesquiterpenes and some long-chain terpenoids, like rubber and dolichols.

The diversity of terpenoid metabolites in plants and the presence of multiple biosynthetic pathways competing for the same prenyl diphosphate substrates suggest that molecular mechanisms must exist to sufficiently direct the metabolic flux toward the formation of specific terpenoid compounds. Recent studies also indicated that some *cis*-prenyl diphosphates could inhibit other terpenoid biosynthetic enzymes (Gutensohn et al., 2014), further highlighting the need for containment and channeling of certain intermediates in the plant terpenoid biosynthetic network. Due to their hydrophobicity, terpenoid metabolites could partition to any cellular membrane, therefore biosynthetic pathways should also involve mechanisms that target respective terpenoid compounds toward specific membranes in the plant cell.

The concept of metabolons, supramolecular complexes of sequential metabolic enzymes and cellular structural elements that mediate substrate channeling, was proposed already more than three decades ago (Srere, 1985). While these multi-protein complexes can be either transient and dynamic, or more stable associations of enzymes (Winkel, 2004; Zhang and Fernie, 2021), a clear picture has emerged about their potential functions (Obata, 2020). They (i) sequester pathway intermediates thus increasing their local concentrations and subsequently enhancing reaction rates, (ii) prevent the release of pathway intermediates thus precluding their potential degradation or harmful reactions with other cellular components, (iii) protect from the access of compounds acting as enzyme inhibitors, (iv) restrict the consumption of intermediates by competing enzymes thus determining the direction of metabolic fluxes, and (v) frequently interact with membranes or cytoskeleton elements thus controlling the localization of enzymes. An increasing number of metabolons has recently been characterized that are involved in plant central and specialized metabolism (summarized in Winkel, 2004; Obata, 2019; Zhang and Fernie, 2021). Here, we summarize the current knowledge and discuss future challenges in studying the role of metabolic channeling and potential metabolons in plant terpenoid metabolism as well as respective applications in the metabolic engineering of terpenoid production.

## GGPPS containing complexes in plastids

In metabolic networks the same precursor is often shared by several biosynthetic pathways localized in the same subcellular compartment, which requires the regulation of its allocation toward each pathway. In the plant terpenoid metabolic network, prenyltransferases catalyze key reactions controlling carbon flux toward the biosynthesis of distinct classes of terpenoid compounds. In plastids, for example, GGPP biosynthesis represents such an essential branch-point as this precursor is shared by several vital pathways including the formation of gibberellins, carotenoids, tocopherols, phylloquinons, plastoquinones, and chlorophylls (Figure 1). Indeed, the Arabidopsis knock-down mutant of a plastidic GGPPS (*ggpps11*), a hub isozyme required for the production of photosynthesis-related metabolites, contains reduced levels of chlorophylls, carotenoids, tocopherols, phylloquinons, and plastoquinones (Ruiz-Sola et al., 2016). It was found that GGPPS11 interacts with three GGPP-utilizing enzymes: geranylgeranyl reductase (GGR) involved in the formation of phytyl chains for chlorophyll, tocopherol and phylloquinone; PSY involved in carotenoid formation; and solanesyl diphosphate synthase (SPS) involved in plastoquinone formation (Ruiz-Sola et al., 2016). These results suggested that multi-enzyme complexes containing GGPPS and GGPP-consuming enzymes could present a mechanism for channeling of metabolic flux toward specific downstream biosynthetic pathways. Characterization of GGPPS in rice chloroplasts provided further details about the molecular mechanisms regulating the allocation of prenyltransferases to such complexes (Zhou et al., 2017). Rice contains only one functionally active GGPPS, OsGGPPS1, which exists as a homodimer in the chloroplast stroma. However, a thylakoid localized GGPPS recruiting protein (GRP) forms a heterodimer with OsGGPPS1 and recruits the latter toward thylakoid membranes. The catalytically inactive GRP not only enhances the catalytic efficiency of OsGGPPS1, but also directs it to a multi-enzyme complex consisting of GGR, light-harvesting-like protein 3 (LIL3), protochlorophyllide oxidoreductase (PORB) and chlorophyll synthase (CHLG). These results suggest that the interaction of GRP with OsGGPPS1 provides a mechanism for recruiting GGPPS toward a thylakoid localized metabolon thus increasing metabolic flux toward chlorophyll biosynthesis and reducing flux toward competing terpenoid pathways. Remarkably, a similar interaction between GGPPS and a GRP homolog has also been demonstrated in ripening fruits of red pepper (*Capsicum annuum*) that accumulate carotenoids (Wang et al., 2018). In *C. annuum* both, GGPPS and GRP, interact with PSY, which previously has been found in a protein complex involved in carotenoid formation (Dogbo and Camara, 1987). Moreover, phytoene desaturase (PDS), the enzyme acting downstream of PSY in the carotenoid biosynthesis, was likewise found to be present in a large protein complex in plastid membranes (Lopez et al., 2008). Thus, it appears that in plastids GGPPS can also be recruited into a membrane-bound protein complex that contains PSY, PDS and potentially other enzymes, thereby directing the metabolic flux toward carotenoid formation.

## Complexes involved in sterol and triterpenoid biosynthesis

Similar to the situation in plastids where multiple biosynthetic pathways compete for GGPP, FPPS resides at a branch-point of the plant terpenoid metabolic network in the cytosol (Figure 1). The synthesized *E*,*E*-FPP is utilized by competing biosynthetic pathways leading to the formation of sesquiterpenes, sterols, brassinosteroids, and triterpenes. Earlier studies showed that the exposure of tobacco and potato to pathogens or elicitors increased the biosynthesis of sesquiterpenes while concomitantly decreasing sterol formation, indicating an altered allocation of *E*,*E*-FPP between these competing pathways (Threfall and Whitehead, 1988; Vögeli and Chappell, 1988; Zook and Kuc, 1991). Squalene synthase (SQS) catalyzing the first committed step of sterol biosynthesis contains an N-terminal catalytic domain and a C-terminal domain tethering the enzyme to the endoplasmic reticulum membrane. A 26-amino acid hinge region linking these two domains was found to be unique to SQSs in different kingdoms of life (e.g., plants and fungi). Expression of Arabidopsis SQS1 in the yeast Δ*erg9* background could only partially complement this SQS deletion mutant, while a chimeric *Arabidopsis* SQS carrying the C-terminus of the yeast enzyme fully restored the fungal sterol formation (Kribii et al., 1997; Linscott et al., 2016). Remarkably, Δ*erg9* yeast lines expressing the plant SQS produced presqualene diphosphate, a toxic intermediate normally not released from the enzyme, suggesting that the SQS hinge region is involved in the interaction with downstream sterol biosynthetic enzyme(s) and crucial for metabolic channeling in this pathway. Moreover, exogenously supplied squalene was a poor substrate for the second pathway enzyme squalene epoxidase (SQE) when analyzed in a yeast microsomal fraction, while *E*,*E*-FPP was efficiently converted to the SQE product 2,3-oxidosqualene (M’Baya and Karst, 1987), supporting the existence of SQS and SQE proximity that allows metabolite channeling.

A complex network of protein–protein interactions among sterol biosynthetic enzymes was also discovered in yeast using yeast two-hybrid analysis (Mo and Bard, 2005) further confirming the involvement of a multi-enzyme complex in this pathway. The core of this complex is found in fungi, humans and plants, and appeared to be formed by three enzymes involved in the sterol C-4 demethylation, namely sterol C-4 methyl oxidase (SMO), sterol C-3 dehydrogenase/C-4 decarboxylase (CSD) and sterol C-3 keto-reductase (SKR), *via* their interaction with the non-catalytic protein Erg28 (Gachotte et al., 2001; Mialoundama et al., 2013). This core complex likely acts as a hub with which enzymes acting earlier in the pathway, such as oxidosqualene cyclase (Erg7p) (Taramino et al., 2010), as well as later pathway enzymes such as Erg6p can interact (Mo and Bard, 2005). Such an organization also allows the variability in the sterol biosynthesis found in different organisms that results in sterol compounds with diverse positions of double bonds and side-chain alkyl groups introduced in the downstream enzymatic steps (Mo and Bard, 2005). The role of the core complex in metabolic channeling in the sterol biosynthesis in plants was highlighted by the fact that RNAi and mutant lines for *Arabidopsis thaliana ERG28* accumulated 4-carboxy-4-methyl-24-methylenecycloartanol (CMMC), a sterol biosynthetic intermediate (Mialoundama et al., 2013). CMMC is channeled within the complex to produce membrane sterols and brassinosteroids under normal conditions, but is released once the complex is deregulated and inhibits polar auxin transport (Mialoundama et al., 2013).

## *cis*-Prenyltransferase complex involved in rubber formation

While over 2500 plant species produce natural rubber, a *cis*-1,4-polyisoprene, only a few have been used to study its biosynthesis in detail (summarized in Cherian et al., 2019) including the para rubber tree, guayule, and rubber dandelion. The basic biochemical mechanism of rubber formation appears to be relatively conserved among these species. It involves *cis*-prenyltransferases (CPTs), also called rubber transferases, that catalyze the sequential *cis*-1,4-condensation of IPP, initially onto *trans*-short chain prenyl diphosphates serving as priming substrates, and then to the α-terminus of polyprenyl pyrophosphates and the partially polymerized rubber molecules. Rubber biosynthesis takes place at the surface of rubber particles that are surrounded by a lipid monolayer membrane with integrated and associated proteins, and contain a hydrophobic core of rubber polymers. While some of the proteins in rubber particles only have structural functions, CPTs form a multi-protein complex, which is responsible for rubber biosynthesis. This rubber transferase complex contains two small substrate-binding proteins (1.6–1.8 and 3.6–3.9 kDa) that bind IPP and FPP (Cornish et al., 2008) and likely initiate the biosynthetic process. The CPTs were found to directly interact with a non-catalytic CPT-binding protein (CBP), which links them to the rubber particle to ensure efficient rubber biosynthesis (Yamashita et al., 2016; Lakusta et al., 2019; Niephaus et al., 2019). Rubber elongation factor (REF) is another protein interacting with CPT (Yamashita et al., 2016), however, its role in rubber biosynthesis is not fully resolved. A small rubber particle protein (SRPP) interacts with REF, thus also representing a subunit of this multi-protein complex, and appears to play a role in regulating the molecular weight of the rubber polymer (Collins-Silva et al., 2012). Overall, the rubber transferase complex represents a metabolon that is involved in substrate binding, catalysis, molecular weight regulation, and particularly in channeling of the hydrophobic polyisoprene product into the interior of the rubber particle.

## Metabolic channeling in the engineering of terpenoid formation

**FIGURE 2:**
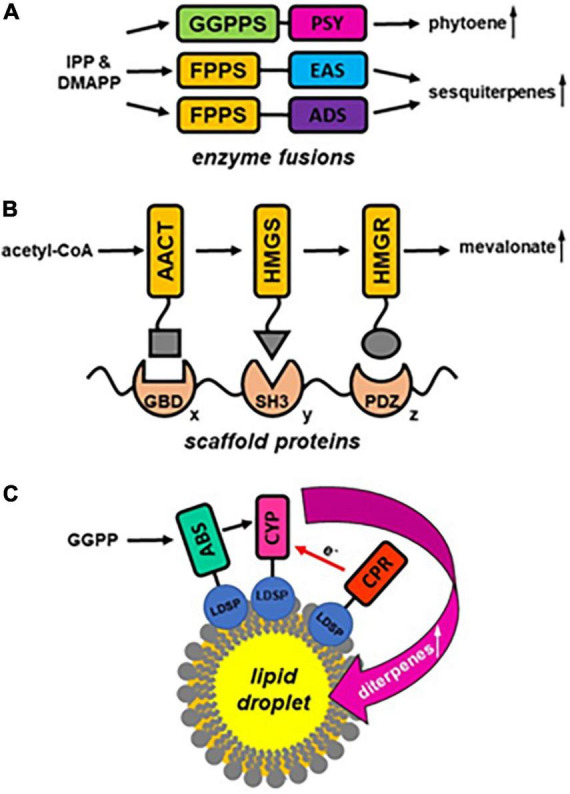
Engineering approaches utilizing metabolic channeling for improved terpenoid formation. **(A)** Enzyme fusion strategy: fusion of prenyltransferases (GGPPS, FPPS) with phytoene synthase (PSY) and terpene synthases (*epi*-aristolochene synthase EAS, amorpha-4,11-diene synthase ADS), respectively, leads to increased substrate (IPP, DMAPP) conversion to product formation. **(B)** Synthetic scaffold protein strategy: scaffold proteins carry multiple protein-protein interaction domains (GBD, SH3, PDZ) in various ratios (x: y: z) to organize multiple sequential pathway enzymes (e.g., AACT, HMGS, HMGR) tagged with specific small peptide ligands (indicated by a square, triangle and circle) to increase metabolic flux through a pathway. **(C)** Lipid droplet strategy: engineered cytosolic lipid droplets serve as scaffold to co-localize terpenoid biosynthetic enzymes, e.g., diterpene synthase abietadiene synthase (ABS), cytochrome P450 (CYP), and cytochrome P450 reductase (CPR), that are expressed as fusion constructs with a lipid droplet surface protein (LDSP). It will result in increased production of terpenoid compounds (e.g., diterpenes) and additionally in their sequestration in the engineered lipid droplets.

The above examples suggest that metabolons are an integral part of terpenoid metabolism in plants that reduce metabolic competition while increasing the flux toward the formation of distinct terpenoid products, prevent the release of potentially harmful pathway intermediates, and direct terpenoid products toward specific membranes and cellular compartments. Thus, it is not surprising that this emerging knowledge was already applied in the metabolic engineering of terpenoid production. Three different strategies have been explored so far to achieve metabolic channeling in engineered terpenoid biosynthetic pathways (Figure 2). The simplest approach to enhance the metabolic flux is to utilize fusions of metabolic enzymes catalyzing successive reactions. To engineer increased carotenoid formation in plants the co-expression of GGPPS and PSY as individual enzymes was compared to the expression of a fusion construct with GGPPS linked to the C-terminus of PSY (Camagna et al., 2019). While the individual enzymes converted only 60% of the IPP substrate to phytoene suggesting significant GGPP leakage, the overexpression of the PSY-GGPPS construct in Arabidopsis (Figure 2A) resulted in almost quantitative conversion of IPP implying efficient metabolite channeling within the enzyme fusion. Likewise, fusion constructs containing FPPS linked *via* a short Gly-Ser-Gly peptide linker to sesquiterpene synthases (Figure 2A), *epi*-aristolochene synthase (EAS) or amorpha-4, 11-diene synthase (ADS), were designed and tested *in vitro* and *in planta* (Brodelius et al., 2002; Han et al., 2016). The fusion of enzymes did not affect their folding and affinity for substrates as the *K*_m_ values were the same for the single FPPS and EAS, as well as EAS-FPPS linked enzymes (Brodelius et al., 2002), but the amount of produced *epi*-aristolochene was significantly higher in case of fused proteins (Brodelius et al., 2002). Similarly, plants expressing the FPPS-ADS fusion construct displayed a higher flux toward artemisinin formation (Han et al., 2016) suggesting that the close proximity of the fused enzymes reduced the diffusion of FPP substrate from the engineered metabolons. Although enzyme fusions can result in substrate channeling, this approach suffers from several restrictions including (i) the potential negative effects on protein folding, (ii) the limitation in the number of enzymes that can be linked, and (iii) the fact that only a fixed 1:1 ratio of enzymes can be achieved due to the fusion. An alternative metabolic engineering approach addresses these issues by utilizing synthetic scaffold proteins to organize multiple enzymes into complexes. For this purpose, scaffold proteins carrying multiple protein-protein interaction domains are used to co-localize multiple sequential pathway enzymes (Figure 2B), each tagged with a specific small peptide ligand that will bind to the domains on the scaffold and is less likely to interfere with protein folding (Lee et al., 2012). The modular architecture of the scaffold not only allows combining more than two enzymes, but also to control the relative ratio of enzymes within the metabolon by varying the number of interaction domains in the scaffold. The feasibility of this approach was tested for three sequential MVA pathway enzymes (Figure 1), acetoacetyl-CoA transferase (AACT), hydroxy-methylglutaryl-CoA synthase (HMGS) and hydroxy-methylglutaryl-CoA reductase (HMGR) (Dueber et al., 2009). Upon optimizing the stoichiometry, this multi-enzyme complex organized by a scaffold resulted in a 77-fold improvement in mevalonate formation and reduced the accumulation of intermediates. A third approach to create a scaffold for multiple terpenoid biosynthetic enzymes involves the engineering of ER-derived cytosolic lipid droplets in plants (Sadre et al., 2019). When the diterpene synthase abietadiene synthase (ABS), cytochrome P450 (CYP), and cytochrome P450 reductase (CPR) were expressed as fusion constructs with a lipid droplet surface protein (LDSP) in *Nicotiana benthamiana* leaves with engineered lipid droplets these proteins all co-localized at the surface of the artificial organelles (Figure 2C). Co-expression of cytosolic HMGR and GGPPS in these leaves resulted not only in the efficient formation of diterpenoids and diterpenoid acids, but also in the sequestration of these products in the engineered lipid droplets which might limit negative feedback on the pathway.

## Conclusion

Metabolons by definition are transient multi-protein complexes of sequential enzymes that mediate substrate channeling (Srere, 1985; Zhang and Fernie, 2021). To date, the presence of many metabolons has been proposed in plants based on observed protein-protein interactions, however, only in a few cases channeling of metabolites has been proven yet (Achnine et al., 2004; Graham et al., 2007; Laursen et al., 2016; Zhang et al., 2017). Here we focused on potential metabolons in the plant terpenoid metabolism, for which just protein interactions and complexes have been demonstrated so far, and only indirect evidence for substrate channeling exists from genetic studies. The formation of metabolons and their dynamic nature could provide an additional level of regulation in the complex and highly branched plant terpenoid metabolic network. Such type of regulation allows to rapidly redirect metabolic fluxes depending on changing demands during a plant’s development or in response to its abiotic and biotic environment. However, to understand the role of metabolons in terpenoid biosynthetic pathways in particular a further detailed analysis of substrate channeling is required and could be achieved by (i) isotope dilution and enrichment studies, (ii) transient time analysis, (iii) evaluating resistance to competing reactions and inhibitors, and (iv) enzyme-buffering analysis (Fernie et al., 2018; Sweetlove and Fernie, 2018). Moreover, future investigation of interactions of terpenoid biosynthetic enzymes at different developmental stages and under changing environmental conditions will uncover potential additional players of novel or currently proposed metabolons. While GGPPS was found to form complexes with various downstream terpenoid biosynthetic enzymes as summarized above, no interactions, for example, were identified yet between FPPS and enzymes of the sterol biosynthesis pathway or cytosolic terpene synthases, despite indications for changing metabolic fluxes between these competing pathways (Threfall and Whitehead, 1988; Vögeli and Chappell, 1988; Zook and Kuc, 1991).

## Author contributions

MG conceived the concept for this manuscript. All authors wrote the manuscript and have read and approved the final manuscript.
